# Optimization of Tris/EDTA/Sucrose (TES) periplasmic extraction for the recovery of functional scFv antibodies

**DOI:** 10.1186/s13568-020-01063-x

**Published:** 2020-07-20

**Authors:** Elham Ghamghami, Marjan Abri Aghdam, Mohammad Reza Tohidkia, Asadollah Ahmadikhah, Morteza Khanmohammadi, Tayebeh Mehdipour, Ahad Mokhtarzadeh, Behzad Baradaran

**Affiliations:** 1 Department of Biological Science, Faculty of Basic Science, Higher Education Institute of Rab-Rashid, Tabriz, Iran; 2 Immunology Research Center, Tabriz University of Medical Sciences, Tabriz, Iran; 3 Faculty of Life Sciences and Biotechnology, Shahid Beheshti University, G.C Velenjak, Tehran, Iran; 4 Chemical Engineering Faculty, Sahand University of Technology, Sahand New Town, Tabriz, Iran; 5 Research Center for Pharmaceutical Nanotechnology, Tabriz University of Medical Sciences, Tabriz, Iran

**Keywords:** TES extraction, Periplasmic extraction, scFv, *E. coli*

## Abstract

Single-chain variable fragments (scFvs) have gained increased attention among researchers in both academic and industrial fields owing to simple production in *E. coli.* The *E. coli* periplasm has been the site of choice for the expression of scFv molecules due to its oxidizing milieu facilitating correctly formation of disulfide bonds. Hence, the recovery of high-yield and biologically active species from the periplasmic space is a critical step at beginning of downstream processing. TES (Tris/EDTA/Sucrose) as a simple and efficient extraction method has been frequently used but under varied extraction conditions, over literature. This study, for the first time, aimed to interrogate the effects of four independent variables (i.e., Tris–HCl concentration, buffer’s pH, EDTA concentration, and incubation time) and their potential interactions on the functional extraction yield of an scFv antibody from the periplasmic space of *E. coli*. The results indicated that the Tris–HCl concentration and pH are the most significant variables in the TES method and displayed a positive effect at their lower values on the functional extraction yield. Besides, the statistical analysis revealed 4 significant interactions between different variables. Here is the first report on the successful application of a design of experiment based on a central composite design to establish a generic and optimal TES extraction condition. Accordingly, an optimal condition for TES extraction of scFv molecules from the periplasm of HB2151 at the exponential phase was developed as follows: 50 mM Tris–HCl at pH 7.2, 0.53 mM EDTA, and an incubation time of 60 min.

## Key points

- Two important variables in TES extraction were Tris–HCl and buffer pH.
- Tris–HCl and buffer pH negatively affected the TES extraction efficiency of scFv.
- The quadratic model developed the optimum conditions for TES extraction.

## Introduction

In the past two decades, different technologies and expression platforms have been used for the successful production of recombinant antibody fragments, including single-chain variable fragment (scFv), fragment antigen binding (Fab), and nanobody, as new classes of therapeutic monoclonal antibodies (Gupta and Shukla 2017). The antibody fragments have great potential applications in molecular biology, and medical diagnosis and therapy (Dangi et al. 2018). Among the antibody fragments, scFv is the well-known and the most extensively used antibody fragment owing to its versatility. The scFv molecule that is composite of the variable domains of heavy chain (V_H_) and light chain (V_L_) covalently connected via a flexible polypeptide linker maintains the entire binding specificity of the conventional whole antibodies (Mohammadi et al. 2016). In contrary to the conventional whole antibody, the small size of scFv molecule (with a molecular weight of approximately 27–30 kDa) provides the advantages of improved tumor penetration, rapid renal clearance and so a short blood half-time, the lower toxicity through reducing off-target effect, cost-effective production, and rarely development of the unwanted immunogenicity arising from anti-drug antibody immune responses, all of which are benefits for medical diagnosis and therapeutic purposes (Xenaki et al. 2017). To date, an increasing variety of expression systems have been developed ranging from bacteria, insects, yeasts, mammalian cells to transgenic plants and animals. Of these expression systems, *E. coli* is the most popular used system for the production of scFv antibody fragments which take advantage of high productivity, straightforward cloning procedures, and the lowest manufacturing costs (Ahmad et al. 2012; Gupta et al. 2017). The scFv fragments often contain two disulfide bonds in their structure and so the correct formation of disulfide bridges in the scFv molecules is substantial for preserving their antigen-binding affinity and stability. The most commonly used expression method for recovering the folded disulfide bond-containing scFvs is to direct them to the oxidizing periplasmic space which takes place between the outer and inner membrane of *E. coli*. (Rodriguez et al. 2017; Sushma et al. 2011). Though, the periplasmic yield of antibody fragments is limited due to small volume of the *E.coli* periplasmic space but production of the scFv molecules in the periplasm offers a great number of advantages including: facilitating disulfide bond formation and proper folding, avoiding the intracellular protein degradation by proteases, and selective releasing the periplasmic scFvs with low host proteins and DNA contaminations resulting in less purification challenges during the product recovery (Gupta and Shukla 2016, 2017; Kasli et al. 2019).

A variety of physical or chemical approaches such as sonication, freeze & thaw, osmotic shock treatment, lysozyme-EDTA treatment, and Tris–HCl/EDTA/Sucrose extraction (TES extraction) have been employed with different degrees of success to recover periplasmic recombinant proteins via disrupting the outer membrane of *E. coli* (Chen et al. 2004; Quan et al. 2013). The periplasmic proteins recovered by these methods have demonstrated different yields and a varied spectrum of cytoplasmic protein contamination depending on the levels of lysis found in outer and/or inner cell membrane (Chen et al. 2004, 2005).

In phage antibody display layouts, osmotic shock treatment and TES extraction have been frequently implemented for the selective release of scFv from the *E. coli* periplasm, leaving the cytoplasmic membrane intact, as a starting material for the purification and in vitro characterization of scFv molecules. Based on a study conducted by S. Quan and colleagues, TES extraction was found to be an ideal method to obtain the cleanest periplasmic and cell envelope proteins (Quan et al. 2013). However, our literature review on phage library-derived scFvs has revealed that diverse conditions have been used for the TES extraction of periplasmic scFvs (Boshuizen et al. 2014; Levy et al. 2013). Accordingly, in the present study, we aimed to develop an optimum and generic approach for the lab-scale TES extraction of scFv antibody fragments from the periplasmic space of *E. coli* HB2151, which is being often used for the expression of soluble scFvs isolated from a phage antibody library. To achieve this, the statistical design of experiment was applied to intensively analyze the effect of different TES extraction conditions on the recovery of a soluble and functional scFv antibody from the periplasm of HB2151*.*

## Materials methods

### Strains and plasmids

The *E. coli* HB2151 strain (K12 *ara ∆(lac-proAB) thi/F' proA* + *B lacIq lacZ∆M15*) harboring a human scFv fragment, which was panned from the Tomlinson I + J library (Source BioScience, Nottingham, UK) against glycine-extended gastrin 17 (anti-G17-Gly) in a previous study (Khajeh et al. 2018), was used for periplasmic extraction experiments. The anti-G17-Gly scFv was expressed through an ampicillin-resistant phagemid vector, pIT2, consisting of IPTG-inducible *lac* promoter, the *pelB* signal sequence for transportation to periplasm space, and c-myc and His tags for its characterization.

### Design of experiment

A design of experiment based on the central composite design (CCD) was employed to systemically analysis the effects of four independent variables presented in TES extraction (i.e., Tris–HCl, EDTA, incubation time and pH), in individual and reciprocal actions, on the recovery yield of the functional anti-G17-Gly scFv from the periplasm of *E. coli* HB2151. The values of independent variables used in the CCD, which were based on available resources and our laboratory experience, were normalized in coded levels of − 1 (lower value of the experimental conditions), 0 (central point condition), and + 1 (higher value of the experimental conditions). The values of independent variables and the relevant coded levels are indicated in Table 1. By considering all of the combinations of four variables and the central condition, to analyze experimental error and check the curvature of responses, a total of 30 experiments consisting of 2^4^_=_16 cube points, 6 replications at the center point, and 8 axial points were employed in this study (Table 2). The data were fitted with the second-order polynomial model by multiple regression techniques as quotation 1:

1 $$y = \beta_{0} + \sum\limits_{i = 1}^{k} {\beta_{i} x_{i} + \sum\limits_{i = 1}^{k} {\sum\limits_{j = 1}^{k} {\beta_{ij} x_{i} x_{j} + \sum\limits_{i = 1}^{k} {\beta_{ii} x_{ii}^{2} } } } }$$

**Table 1:** Experimental deign levels. Values of independent variables and corresponding coded levels were used in a central composite design

Symbols	Variables	Real values of coded levels		
		− 1	0	+ 1
A	Tris (mM)	50	125	200
B	EDTA (mM)	0.1	1.05	2
C	pH	7.2	8.1	9
D	Time (min)	15	37.5	60

The central point (0) mentions middle values interval between the − 1 and + 1 of each variable

**Table 2:** The extraction yield of anti-G17-Gly scFv (mg/L) for a central composite design comprising 4 variables

Run	Tris (mM)		EDTA (mM)		pH		Time (min)		Anti-G17-Gly scFv (mg/L)
1	50	− 1	1.05	0	8.1	0	37.5	0	1.47
2	50	− 1	2	+ 1	9	+ 1	60	+ 1	1
3	200	+ 1	1.05	0	8.1	0	37.5	0	0.34
4	50	− 1	0.1	− 1	7.2	− 1	60	+ 1	3.0
5	125	0	2	+ 1	8.1	0	37.5	0	0.31
6	50	− 1	0.1	− 1	9	+ 1	60	+ 1	3.16
7	125	0	1.05	0	8.1	0	37.5	0	0.39
8	50	− 1	2	+ 1	7.2	− 1	60	+ 1	3.3
9	125	0	1.05	0	8.1	0	37.5	0	0.415
10	200	+ 1	2	+ 1	9	+ 1	15	− 1	0.58
11	125	0	1.05	0	8.1	0	37.5	0	0.414
12	125	0	1.05	0	7.2	− 1	37.5	0	2
13	200	+ 1	2	+ 1	7.2	− 1	60	+ 1	0.64
14	200	+ 1	0.1	− 1	9	+ 1	15	− 1	0.18
15	50	− 1	0.1	− 1	9	+ 1	15	− 1	1.65
16	50	− 1	2	+ 1	7.2	− 1	15	− 1	2.17
17	200	+ 1	0.1	− 1	7.2	− 1	15	− 1	0.43
18	50	− 1	2	+ 1	9	+ 1	15	− 1	0.77
19	200	+ 1	2	+ 1	7.2	− 1	15	− 1	0.9
20	125	0	1.05	0	8.1	0	37.5	0	0.412
21	125	0	1.05	0	8.1	0	37.5	0	0.4
22	50	− 1	0.1	− 1	7.2	− 1	15	− 1	2.54
23	125	0	1.05	0	9	+ 1	37.5	0	0.3
24	200	+ 1	2	+ 1	9	+ 1	60	+ 1	0.39
25	200	+ 1	0.1	− 1	7.2	− 1	60	+ 1	0.3
26	125	0	1.05	0	8.1	0	15	− 1	1
27	200	+ 1	0.1	− 1	9	+ 1	60	+ 1	0.45
28	125	0	1.05	0	8.1	0	37.5	0	0.42
29	125	0	0.1	− 1	8.1	0	37.5	0	0.42
30	125	0	1.05	0	8.1	0	60	+ 1	0.68

where *y* is a response variable of protein ELISA, *β*_*0*_ is the intercept, *β*_*i*_ are regression coefficients for linear effects, *β*_*ii*_ are the regression coefficients for quadratic effects, *β*_*ij*_ are interaction regression coefficients, and x_i_ is an independent variable.

### Expression of the periplasmic scFv for analysis of TES extraction conditions

The *E. coli* HB2151 cells harboring the anti-G17-Gly scFv phagemid were initially grown overnight at 37 °C on TYE plate supplemented with 2% glucose and 100 μg/mL ampicillin. A single colony from the TYE plate was inoculated into 2xYT containing 2% glucose and 100 μg/mL ampicillin (2xYT-Glc-Amp) and cultured overnight at 37 °C, shaking at 160 rpm. Then, the overnight culture was diluted in 1:100 ratio by 350 mL of fresh 2xYT-0.1% Glc-Amp medium in a 2 L culture media bottle and incubated at 37 °C until reached an optical density at 600 nm (OD_600_) of 0.9. The induction of anti-G17-Gly scFv expression was performed with IPTG at a final concentration of 1 mM at 30 °C for 5 h through the experiment, unless otherwise specified (Fahimi et al. 2018; Fouladi et al. 2019).

At the end of the induction phase, the bacterial cells were harvested from 15 ml of the culture medium in the 15 mL tubes by centrifugation at 2500 g for 10 min at 4 °C. After discarding the supernatants, the cell pellets were resuspended in 0.5 mL of the ice-cold TES buffer having different conditions. As presented in Table 1, to optimize TES extraction conditions, various concentrations of Tris–HCl and EDTA at different pH values with the constant amount of sucrose (20% w/v) were applied. After incubation on ice for different times including 15, 30, and 60 min, the suspensions were centrifuged at 20,000 g, 4 °C for 30 min. The supernatants referred to soluble periplasmic scFv were harvested and stored at − 20 °C until analysis of the functional recovery yield by ELISA assay.

### Purification of anti-G17-Gly scFv

For purification, the anti-G17-Gly scFv clone was expressed as mentioned above except that induction time was prolonged overnight (12 h). The bacteria cells were harvested by centrifugation at 2500 g, 4 °C for 10 min and, subsequently, incubated with 1/20 volume of the initial culture volume of TES buffer (100 mM Tris–HCl pH 8.0, 1 mM EDTA, and 20% sucrose) supplemented with cOmplete ULTRA protease inhibitor cocktail tablets (Roche, Basel, Switzerland) on ice for 30 min. The supernatant containing scFvs was collected by centrifugation at 20,000 g for 30 min at 4 °C and dialyzed against PBS at 4 °C overnight using a 12,400 Da cut-off dialysis tubing cellulose membrane (Sigma-Aldrich Co., Taufkirchen, Germany).

The purification of His-tagged anti-G17-Gly scFv was performed by immobilized metal affinity chromatography (IMAC) using TALON™ resin (Clontech Laboratories, Inc. Mountain View, CA, USA) according to the manufacturer’s instruction. Briefly, 1 mL of the resin slurry was equilibrated with the 10-bed volumes of wash buffer (50 mM Na3PO4 and 300 mM NaCl, pH 7), and then the dialyzed sample of anti-G17-Gly scFv was incubated with the equilibrated resin on a rotator for 20 min at room temperature. Following twice washing with the 10-bed volumes of wash buffer by centrifugation at 1500 g for 10 min, the scFv-resin complex was passed through a 5 mL chromatography column and the resin-bound scFv molecules were eluted using the wash buffer containing 150 mM imidazole. Lastly, the eluted fractions containing scFv were collected and dialyzed against PBS using the Maxi Pur-ALyzer Dialysis tube with 12,000 Da cut-off (SigmaAldrich CHEMIE GmbH, Steinheim, Germany). The total concentration of the purified scFv was measured via following formula: concentration (mg/mL) = OD_280_ nm × M.W. / ε (Rouet et al. 2012), in which M.W. (molecular weight) and ε (the molar extinction coefficient, 1 mg/mL) for anti-G17-Gly scFv were calculated by online ProtParam tool (https://web.expasy.org/protparam/).

### ELISA assay

High-binding 96-well ELISA plates (Biomat) were coated with biotinylated G17-Gly peptide (pEGPWLEEEE-K-s–s-biotin, where pE means pyroglutamic acid) indirectly through the biotin-streptavidin system (Tohidkia et al. 2017). Initially, biotinylated-BSA (Thermo Fisher Scientific, Waltham, MA) was applied to each well at a concentration of 2 μg/mL in phosphate-buffered saline (PBS) and incubated overnight at 4 °C. After 3 washes in PBS containing Tween-20 (PBST, 0.1% v/v), 100 μL/well streptavidin (Bio Basic) at a concentration of 10 μg/mL in PBS was added to the wells and incubated for 90 min at room temperature with gentle shaking. Following three times washing with PBST, the biotinylated peptide was added to the wells (100 μL/well) at a final concentration of 200 nM and then the plates were further incubated for 90 min under gentle shaking at room temperature. After blocking with 2% MPBS (i.e., 2% w/v skimmed milk powder in PBS) for 1 h at room temperature, the plates were incubated with the periplasmic scFv samples prepared in 2% MPBS buffer for 90 min. Detection of antigen-binding scFv fragments was performed using 1:5000 dilution of L-HRP (Thermo Fisher Scientific), which recognizes the variable light chain (kappa chain) of scFv fragments. Finally, the plates were stained by adding 100 μL of 3,3′,5,5′-tetramethylbenzidine (TMB) substrate, and then the reaction progress was stopped with 50 μL of 5% sulfuric acid. The OD values were read at 450 nm and 630 nm, as a reference wavelength for the background subtraction, using a microtiter plate reader (BioTek ELx800, BioTek, and Winooski, VT). The assay was done in duplicate and the blank wells (containing biotinylated BSA and streptavidin without the peptide antigen coating) were also included to evaluate the standard of deviation (% CV) and specificity for validation of the test.

### SDS-PAGE and Western blotting

Expression and purification processes of the soluble periplasmic scFv were visualized by SDS-PAGE and immunoblotting. The samples were prepared in a 5X SDS-sample buffer and boiled for 5 min at 95 °C and then was separated on two 12% SDS-PAGE at 200 V. The one gel was stained using Coomassie Brilliant Blue G-250 and the other one used for blotting on nitrocellulose membrane and immunostaining with anti-c-myc antibody 9E10 (sc-40, Santa Cruz Biotechnology, Santa Cruz, CA, USA) and HRP-conjugated goat anti-mouse IgG (Thermo Fisher Scientific, Waltham, Massachusetts, USA) at a dilution of 1:1000 and 1:3000, respectively.

### Statistical analysis

The statistically significant effects of each variable and the related interactions on the recovery yield of the functional scFv were confirmed by the Student’s F-test using analysis of variance (ANOVA), and a p-value less than 0.05 (*p* < 0.05) was statistically significant. All experimental design and statistical analysis were done using Design-Expert® software version 7 (Stat-Ease, Inc. Minneapolis).

## Results

### Purification and ELISA results

**Fig. 1:**
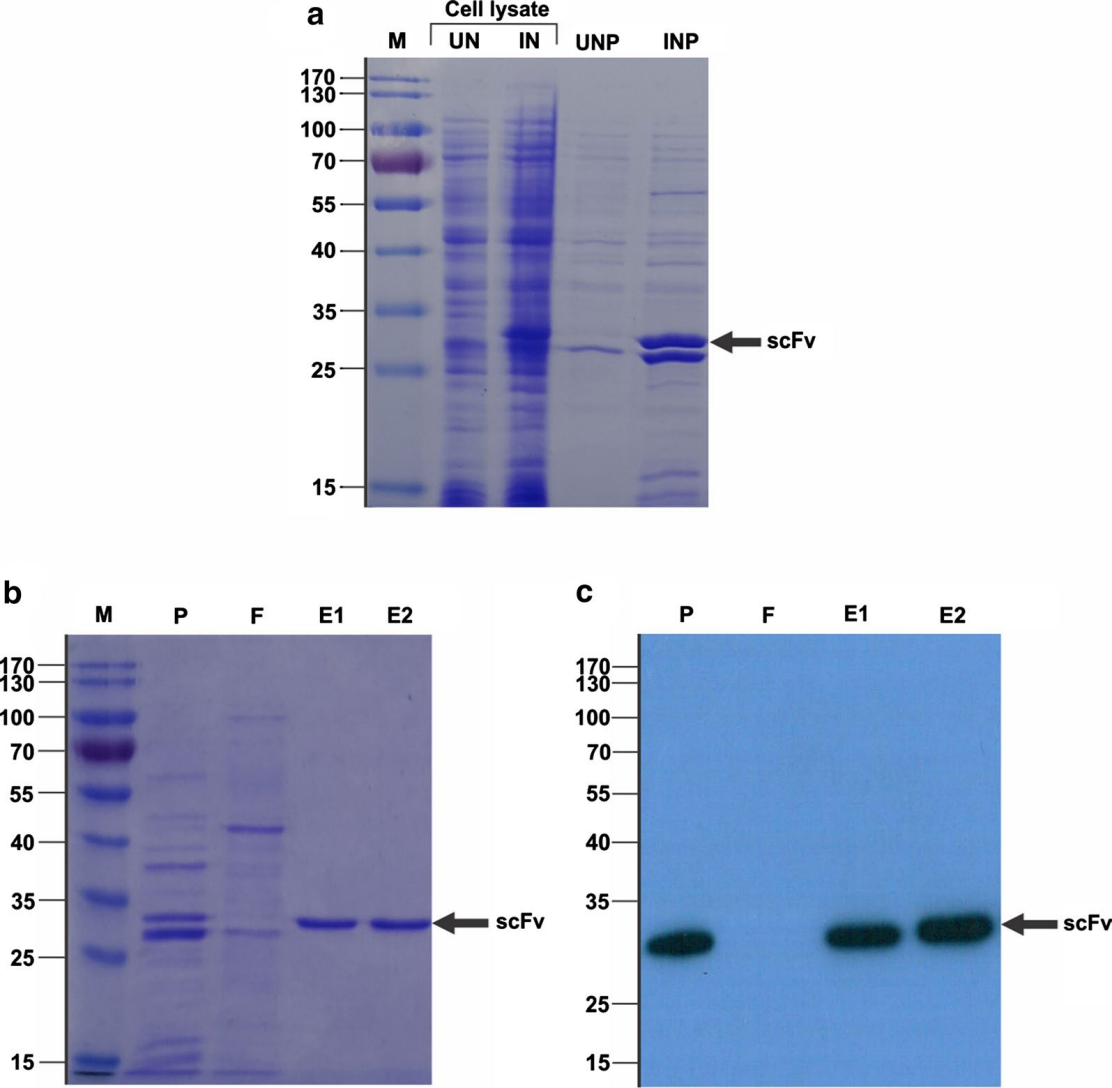
Expression and purification analysis. **a** Shows a sodium dodecyl sulfate–polyacrylamide gel (SDS − PAGE) analysis to confirm the expression of anti-G17-Gly. The identical amount of samples including UN, uninduced total cell lysate; IN, induced total cell lysate; UNP, uninduced periplasm fraction; and INP, induced periplasmic fraction were separated on 12% SDS-PAGE and then stained with Coomassie Brilliant Blue R250. **b**, **c** Show confirmation of the purification process by 12% SDS-PAGE and immunoblotting, respectively. The blots were probed by anti‐c‐myc mAb (9E10) and horseradish peroxidase (HRP)‐conjugated goat anti‐mouse IgG, consecutively. The protein bands were appeared on X‐ray film by incubation with ECL substrate. The arrowheads denote the approximate molecular weight of the scFvs (around 28 kDa). *M* Molecular weight standards in kDa, *P* periplasmic extraction, *F* flow‐through, *E1* and *E2* eluted fractions

The anti-G17-Gly scFv was subjected to express overnight in a shake flask to produce an adequate amount of reagent as the standard control for the following analyses. The presence of a clear and consistent protein band, equivalent to the expected molecular weight of  ~ 28 kDa, in both induced the total cell lysate and periplasmic samples demonstrated successful expression of the anti-G17-Gly scFv (Fig. 1a). Likewise, as shown in Fig. 1b c, the purification process was verified by visualization of the intact and highly pure scFv (above 90%) within the expected molecular size range of scFv molecules. The overall expression yield of anti-G17-Gly scFv was found to be about 10 mg/L of the culture medium.

**Fig. 2:**
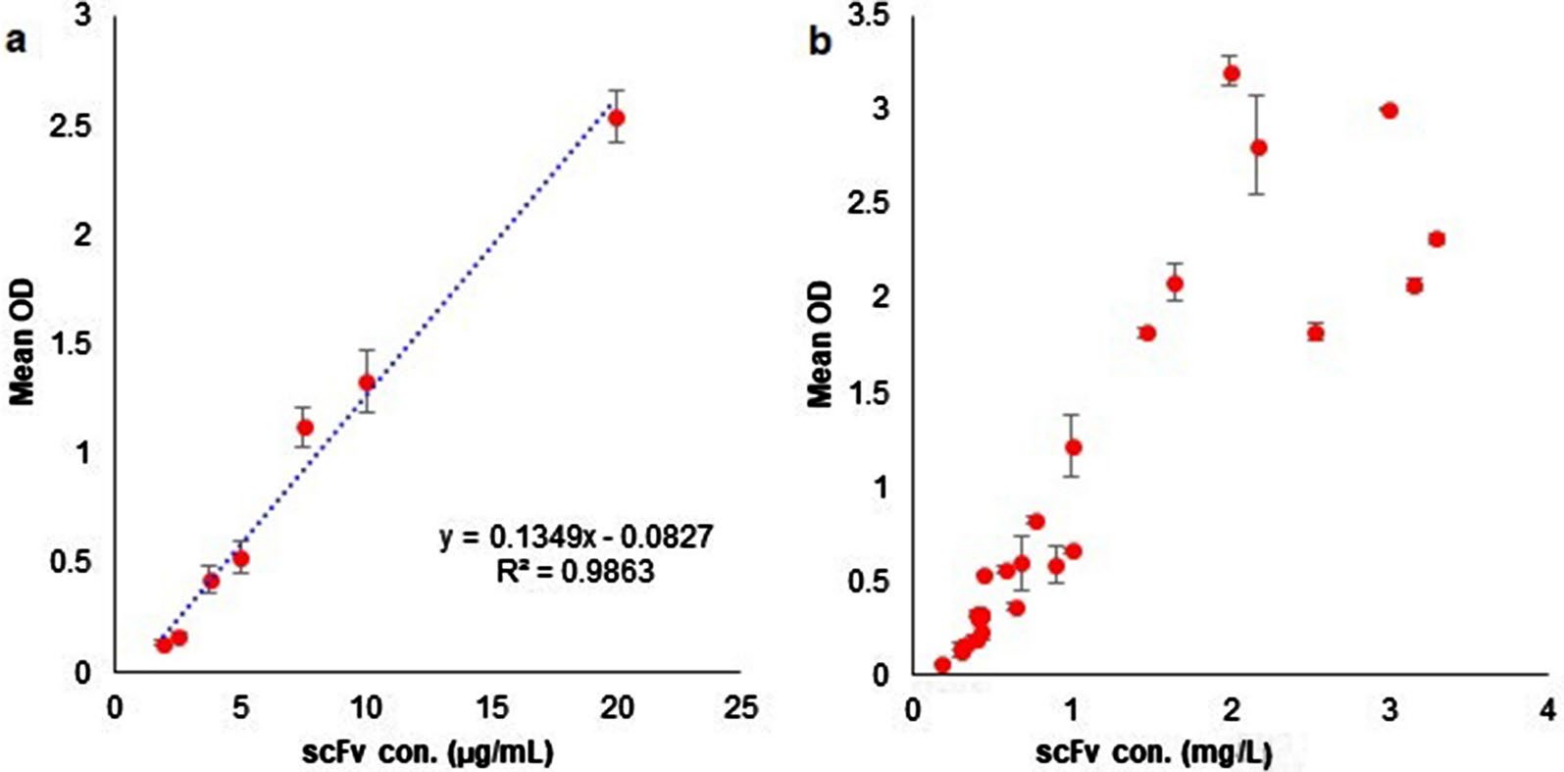
ELISA analysis. **a** Shows a calibration curve constructed by twofold dilution series of the purified anti-G17-Gly. The absorbance values and the standard scFv concentrations showed a satisfactory linear correlation considering R^2^ > 0.9. **b** Represents the overall concentration of the anti-G17-Gly scFv extracts under different experimental conditions. The mean absorbance values of the test samples were interpolated to the linear regression equation obtained from the calibration curve. The Mean OD stands for the average absorbance values of duplicate samples read at 450 nm and 630 nm to subtract background absorbance

The purified anti-G17-Gly scFv with a defined concentration was used for the preparation of standard concentrations through twofold serial dilutions. Then, the calibration curve was constructed by plotting the mean absorbance values of each standard concentration sample with a defined concentration (y-axis) against the corresponding concentrations (x-axis) and choosing the best fit curve for the data points. The calibration curve displayed good linearity with a correlation of determination (R^2^) above 0.98 and the coefficient of variance (CV) less than 14%. The high values obtained for R^2^ suggests a strong linear relationship between the mean absorbance values and the standard scFv concentrations (Fig. 2a). The mean absorbance values of the test samples were interpolated to the calibration curve to calculate the concentration of the anti-G17-Gly scFv extracted under different experimental conditions. The standard deviation of each scFv concentration obtained from duplicate measurements was < 0.13 (Fig. 2b).

### The analysis of experimental design and modeling

To achieve the optimal TES buffer condition, response surface methodology (RSM) using a central composite design was established to investigate the individual and interaction effects of different buffer compositions on anti-G17-Gly scFv extraction from the periplasm of *E. coli* HB2151. A panel of experimental design was produced based on the four variables: Tris–HCl concentration (50–200 mM), EDTA (0.1–2 mM), pH (7.2–9), and incubation time (15–60 min) as indicated in Table 1. Accordingly, 30 experimental conditions were run to examine the functional extraction yield of periplasmic scFv by ELISA. The result showed that the functional extraction yield of periplasmic scFv was ranged from 0.18 to 3.3 mg/L in the different conditions tested (Table 2). The experimental error and reproducibility were evaluated at the central point conditions of the experimental design (125 mM Tris–HCl, 1.05 mM EDTA, pH 8.1, and 37.5 min incubation time) with 6 independent replications. At these conditions, the recovery yield of anti-G17-Gly scFv was 0.41 mg/L ± 0.01 with the coefficient of variation being less than 5%., which defined as the ratio of the standard deviation to the average of anti-G17-Gly scFv concentration (mg /L).

The adopted design of experiment represented the response surface quadratic model to evaluate the effects of variables on the efficacy of scFv extraction. There are a number of statistical analyses demonstrating the excellent adequacy and significance of the quadratic regression model. "Adeq Precision" was used to evaluate the signal to noise ratio of the model showed a high value of 13.466, indicating an adequate and desirable signal. As described in Table 3, The ANOVA analysis demonstrated that the quadratic model developed from RSM was significant with the *F*value of 13.48 and a very low p-value (Prob > F) of < 0.0001 as there was only a 0.01% chance that a "Model *F* Value" could occur because of noise.

**Table 3:** ANOVA for response surface quadratic model

Source	Sum of squares	df	Mean square	*F* value	*p* value Prob > *F*	
Model	23.90	14	1.71	13.48	*< 0.0001*	Significant
A-Incubtime	0.4	1	0.4	3.20	0.094	
B-EDTA	0.24	1	0.24	1.88	0.191	
C-Tris	12.25	1	12.25	96.74	*< 0.0001*	Significant
D-pH	2.57	1	2.57	20.28	*0.0004*	Significant
AB	0.09	1	0.09	0.71	0.413	
AC	0.83	1	0.83	6.54	*0.022*	Significant
AD	0.024	1	0.024	0.19	0.669	
BC	1.13	1	1.13	8.96	*0.009*	Significant
BD	0.74	1	0.74	5.84	*0.029*	Significant
CD	0.83	1	0.88	6.98	*0.019*	Significant
A^2^	0.15	1	0.15	1.15	0.301	
B^2^	0.15	1	0.15	1.16	0.299	
C^2^	0.24	1	0.24	1.86	0.192	
D^2^	0.77	1	0.77	6.12	0.026	
Residual	1.90	15	0.13			
Pure error	6.315E−0.04	5	1.263E−0.04			
Cor total	25.80	29				

**Fig. 3:**
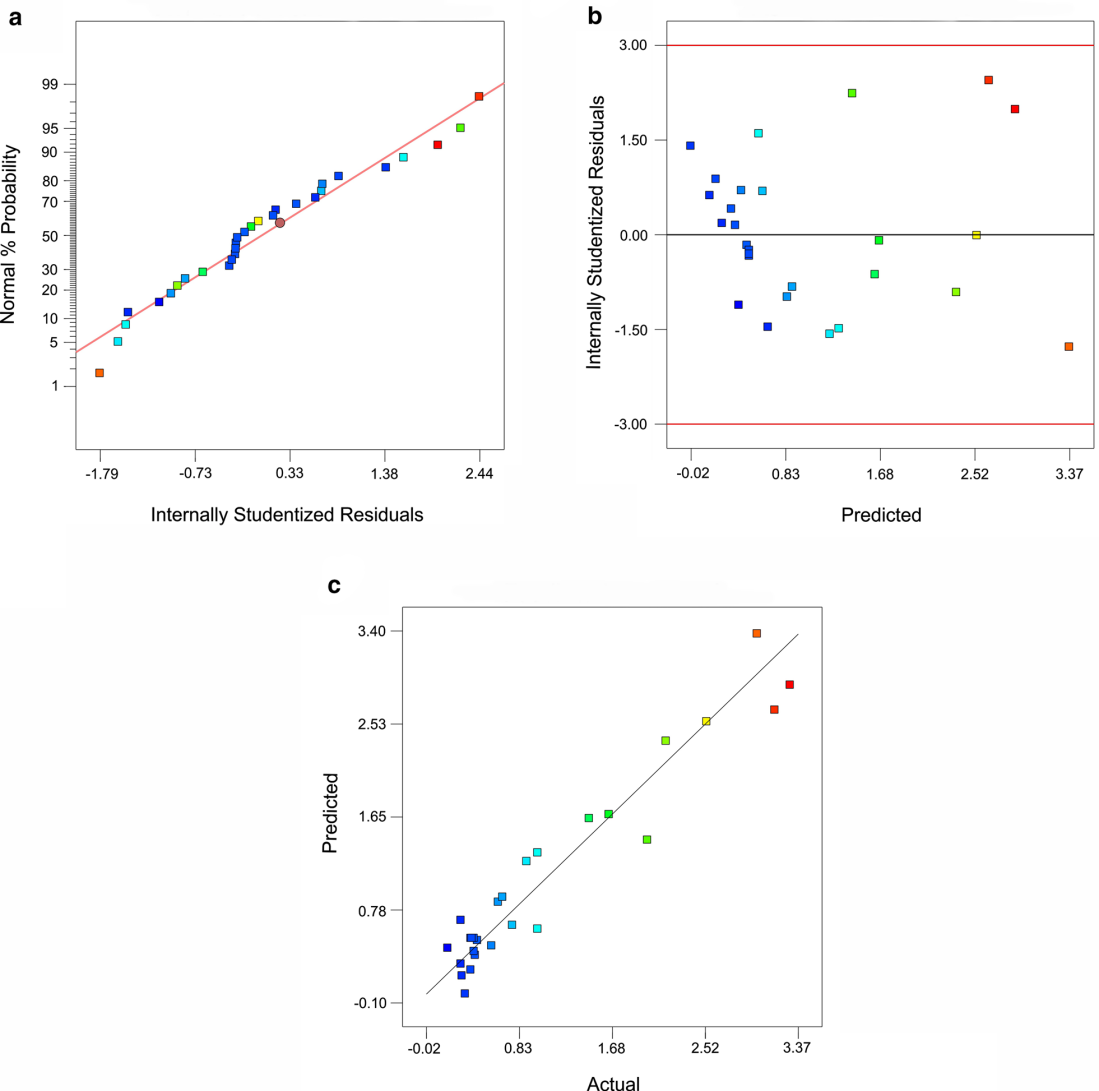
The studentized residual plots for the quadratic model adequacy. The normal plot of residuals (**a**), the plot of internally studentized residuals *vs* predicted response (**b**), and the plot of internally studentized residuals *vs* actual (**c**)

To further authentication of the adequacy of the response surface quadratic model, the studentized residuals measuring the number of standard deviations dividing the model estimated and the experimental values were used. The normal probability plot of anti-G17-Gly scFv extractions was normally distributed upon linear behavior of data points, indicating no deviation of the variance (Fig. 3a). The initial assumption of constant variance which is crucial for a desirable regression model was demonstrated by a random residuals distribution versus the respective predicted values (Fig. 3b). Besides, an excellent agreement between actual and predicted values was observed (Fig. 3c). Overall, the significance and outstanding adequacy of the quadratic regression model was well demonstrated.

### Effects of the variables on TES extraction efficiency

The following quadratic equation adapted from the response surface quadratic model represents a correlation of the defined variables and anti-G17-Gly scFv concentration (mg/L) as the functional extraction yield of the periplasmic scFv.

ScFv extraction (mg/L) =  + 0.51 + 0.15A−0.12B−0.83C−0.38D−0.075AB−0.23AC + 0.039AD + 0.27BC−0.22BD + 0.23CD + 0.24A^2^−0. 24B^2^ + 0.3C^2^ + 0.55D^2^.

The statistical significance of the response surface quadratic equation was confirmed by an F-test and the ANOVA analysis. As depicted in Table 3, the ANOVA analysis revealed that Tris–HCl (C) and pH (D) as the linear terms, as well as, the interaction terms including time/Tris–HCl (AC), EDTA/pH (BD), EDTA/Tris–HCl (BC), and Tris–HCl/pH (CD) had a statistically significant influence on the recovery yield of scFv (*P* < 0.05). Both the Tris–HCl concentration and pH value had significant and negative effects (*P* < 0.0001 and *P* = 0.0004, respectively), indicating that lower Tris–HCl concentrations and pH values enhanced the extraction yield of the periplasmic scFv. However, two other linear terms, the incubation time (A) and EDTA concentration (B), did not show a significant influence on the extraction yield of scFv (*p* > 0.05). In the case of interaction terms, while AC and BD had a significant negative effect with the *p* values of 0.022 and 0.029, respectively, two other model terms of BC (*p* = 0.009) and CD (*p* = 0.019) showed significant positive effects.

**Fig. 4:**
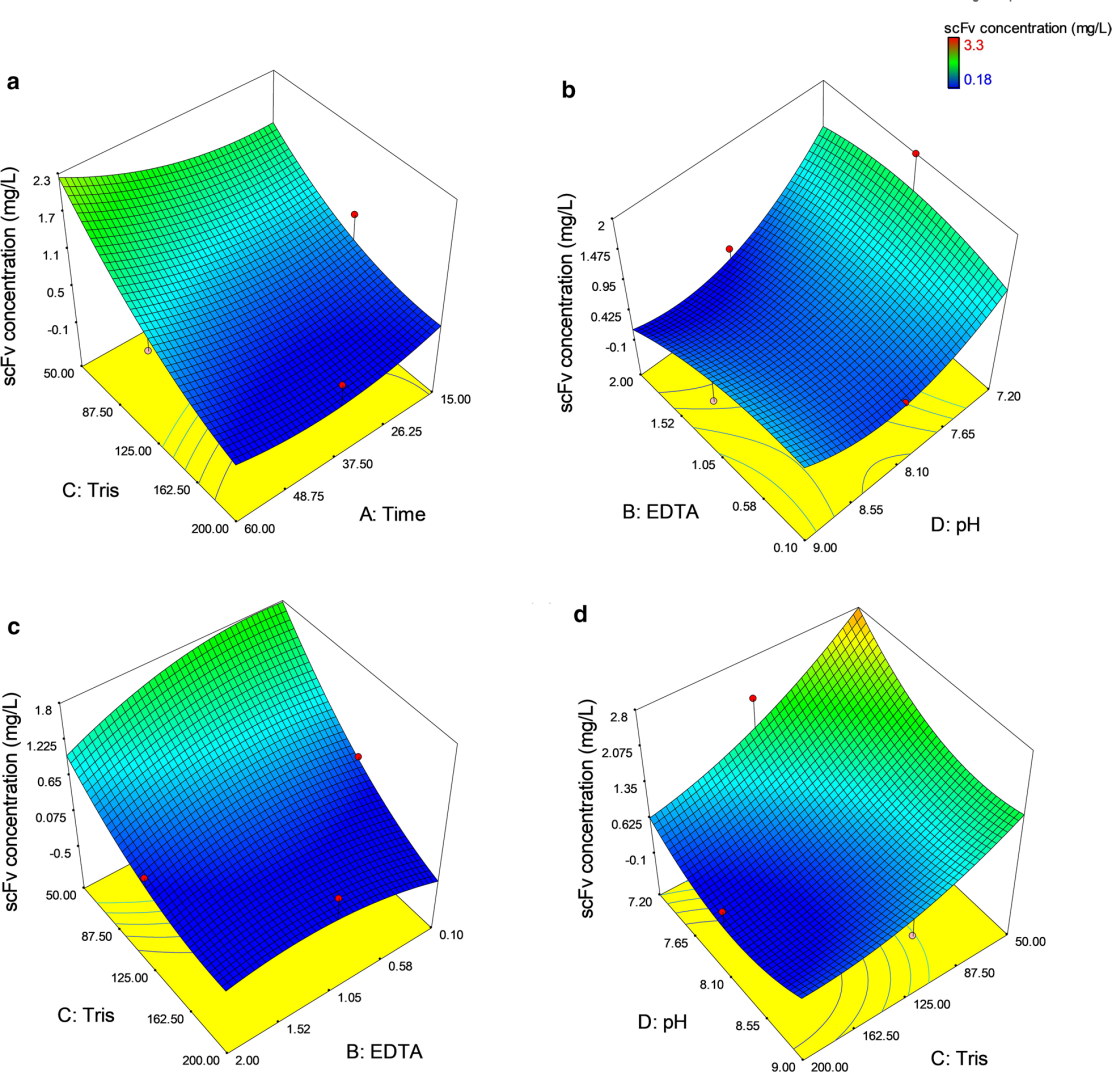
Response surface plots associated with the extraction yield of periplasmic anti-G17-Gly scFv. The statistically significant interaction terms effective in the anti-G17-Gly scFv extraction (mg/L) are showed by the response surface plots: **a** The interaction between incubation time and Tris–HCl concentration (AC); **b** The interaction between EDTA and pH (BD); **c** The interaction between EDTA and Tris–HCl concentration (BC); **d** The interaction between Tris–HCl concentration and pH (CD)

The effects of statistically significant interaction model terms were also represented by 3D response surface plots (Fig. 4). The extraction yield of anti-G17-Gly scFv was negatively affected by the incubation time and Tris–HCl concentration interaction (AC) such that the highest extraction yield was observed at the lower concentrations of Tris–HCl, in particular, at longer incubation time (Fig. 4a). In other words, the extraction yield was more dependent on Tris–HCl concentration than incubation time as confirmed by *p* = 0.0001 *vs p* = 0.094. As illustrated in Fig. 4b, the negative effect of interaction between EDTA concentration and pH interaction (BD) on the extraction yield was also observed in such way that the extraction yield was highly influenced by decreasing in the pH value rather than altering the EDTA concentration (*p* = 0.0004 *vs p* = 0.0.191), representative of the minimum dependency of the extraction yield on the EDTA concentration. The significant dependency of the extraction yield on the interaction between the concentration of EDTA and Tris–HCl (BC) was shown in Fig. 4c. The Tris–HCl concentration was found to be highly influential on the extraction yield mainly at lower concentrations of EDTA. Lastly, the response surface plot related to the interaction between Tris–HCl concentration and pH (CD) was indicated that higher values of buffer’s pH accompanied with higher concentrations of Tris–HCl resulted in lower extraction yield (Fig. 4d).

### Optimum conditions for TES extraction

**Fig. 5:**
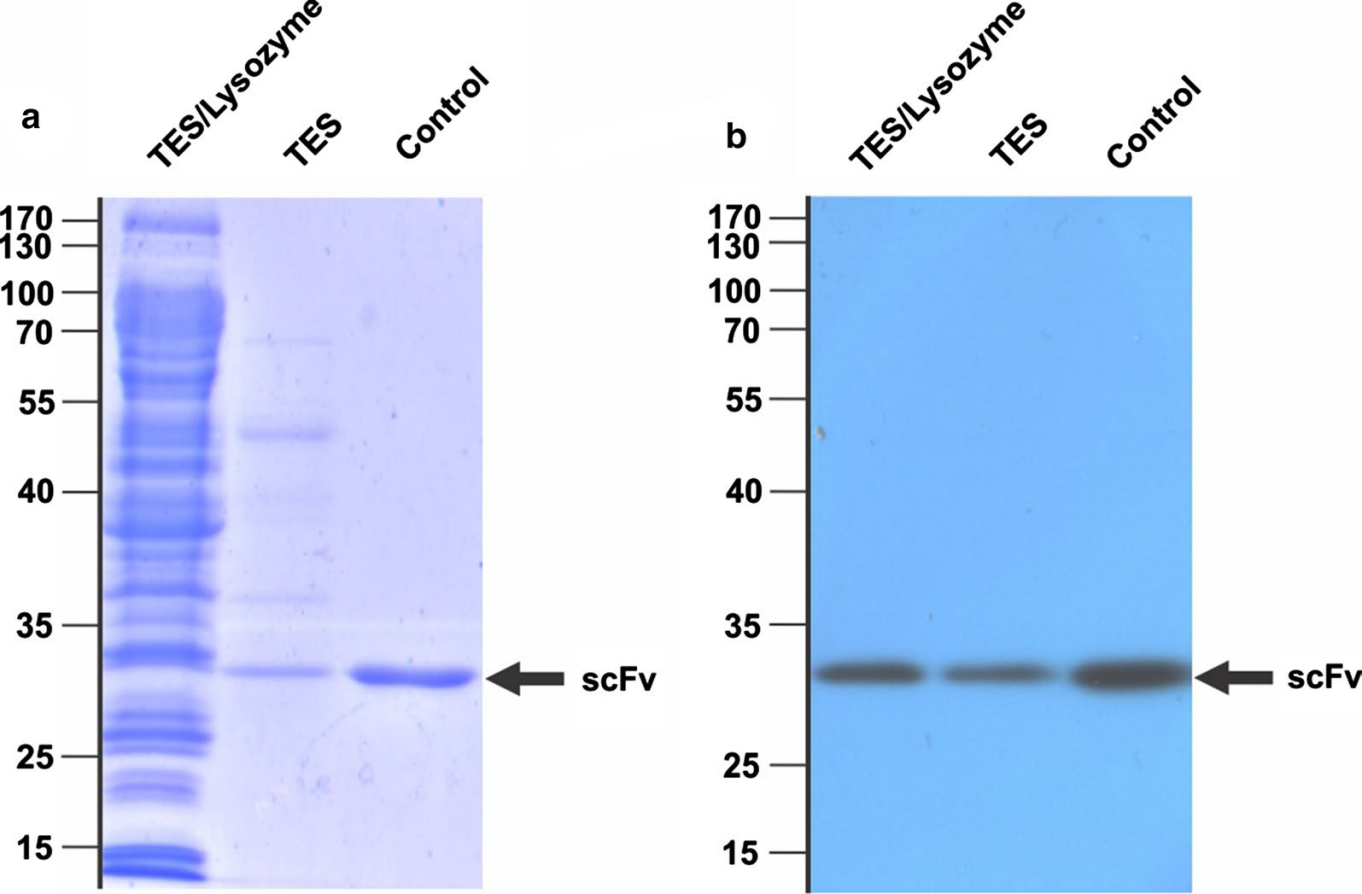
Characterization of the optimal conditions. **a**, **b** display respectively SDS-PAGE and immunoblotting of the anti-G17-Gly scFv expressed at the mentioned optimal conditions. TES extraction shows much purer periplasmic fraction than the TES/lysozyme method. Control is the purified anti-G17-Gly scFv

To interrogate the optimum TES extraction conditions, 30 experimental runs were established by RSM central composite design. The quadratic model developed by design-expert software predicted the optimum buffer conditions for TES extraction, including 50 mM Tris–HCl at pH 7.2, 0.53 mM EDTA, and 60 min incubation time at the constant concentration of sucrose (20%). The optimum conditions produced the highest recovery yield of functional anti-G17-Gly scFv (3.42 mg/L). Experimental validation was performed under the same optimum conditions of TES extraction to estimate the process variability. The functional recovery yield of 3.5 mg/L under the validation test was in an appropriate agreement with the predicted optimal condition and also showed a significant increase around ninefold when compared with central condition points (i.e., 125 mM Tris–HCl at pH 8.1, 1.05 mM EDTA, and an incubation time of 37.5 min). As shown in Fig. 5, SDS-PAGE and immunoblotting analysis of the experimental validation confirmed the expression of anti-G17-Gly scFv with the molecular weight of around 28 kDa corresponding to the predicted size. The TES extraction prepared periplasmic extraction with less host-cell protein contamination when compared to the combined TES/lysozyme extraction method.

## Discussion

Non-amber suppressor strain of *E. coli* HB2151 harboring a recombinant phagemid-scFv represents the most frequently used expression system for the periplasmic expression of the scFv antibody fragments during preliminary studies such as screening, and characteristic (affinity and specificity) and functional analysis, in academic researches (Petrus et al. 2019). The merit of periplasmic expression of scFv molecules possessing disulfide bonds in their structures over other modes of expression can be attributed to the presence of an oxidative environment in the periplasm facilitating the correct disulfide bond formation and proper folding, to the reduced proteolysis of scFvs due to having less periplasmic protease (only 7 out 25 known cellular proteases), at avoiding scFv molecules from the denaturing effect of the air–liquid interfaces, and to less host-cell impurities (DNA, host-cell proteins, and proteases) providing a substantial purification at start of the downstream process (Beacham 1979; de Marco 2009; Gupta and Shukla 2017). The selective release of periplasmically expressed scFvs is the first critical step in the onset of the downstream process. The selective release of periplasmically expressed scFvs, which is undertaken through disrupting or permeabilizing the outer membrane without compromising the integrity of the inner membrane, is of great interest, as it simplifies and increases the effectiveness of downstream purification requirements. TES extraction is a frequently used method which works efficiently on a laboratory scale for the selective release of scFv molecules from the periplasm of *E.* coli HB2151. This method extracts periplasmic fractions much purer than other methods (Tao 2008; Thein et al. 2010) in a simple, inexpensive, and fast fashion, but there have been found diversity in values of the TES buffer composition over the phage antibody display-related literature (Eisenhardt et al. 2007; Mesgari-Shadi and Sarrafzadeh 2017; Noguchi et al. 2017).

In the present study, we aimed to introduce a generic and optimized TES extraction method for the selective recovery of functional scFv antibody fragments from the periplasm space of *E. coli* HB2151 after 5 h induction time at exponential growth phase. For this purpose, four major variables (i.e. Tris–HCl and EDTA concentrations, incubation time, and buffer’s pH) at three levels (Table 1) were considered to investigate their effects on the anti-G17-Gly scFv extraction from the periplasmic space*.* To the best of our knowledge, this study is the first report that interrogates appropriate TES buffer conditions to improve extraction efficiency and protein stability. Unlike most similar researches on the optimization of different extraction methods which used the classical method through changing one variable at a time while keeping other variables constant, here, we used a design of experimental based on RSM/CCD. This multivariate approach allows analyzing the effects of more than one variable at the same time and identifying possible interactions by conducting fewer experiment numbers. Moreover, instead of broadly used SDS-PAGE analysis, the ELISA assay was used in this study to estimate the recovery yield of the periplasmic scFv as a response. The ELISA assay is a more reliable assessment since it represents the functional activity of properly folded scFv molecules which are located at the periplasm space and formed disulfide bounds correctly, as a result, it allows to exclude the unfolded/misfolded fraction of cytoplasmic or even periplasmic scFvs from the analysis (Heo et al. 2006; Su et al. 2003).

Therefore, 30 experimental runs were produced utilizing the CCD method (Table 2) and the response surface quadratic model was adapted to evaluate the effects of variables and their interactions on the functional recovery yield of the anti-G17-Gly scFv. The adequacy and significance of the model were demonstrated by a very low *p*-value Prob > F (< 0.0001) (Table 3) and an excellent agreement between actual and predicted values (Fig. 3c). The adopted model predicted the optimal conditions for TES extraction of the periplasmic scFv as follows: 50 mM Tris–HCl at pH 7.2, 0.53 mM EDTA, and induction time of 60 min. The validation of the experiment performed at these optimal conditions resulted in the recovery yield of 3.5 mg/L, which is consistent with the predicted optimal value of 3.42 mg/L.

The surface response plots and statistical analysis demonstrated that lower concentrations of Tris–HCl strongly increased the functional recovery yield of the anti-G17-Gly scFv when accompanied with a longer incubation time of 60 min, suggesting their significant interaction (Fig. 4a). It was declared that EDTA concentration did not have a significant effect (*p* > 0.05) on the functional recovery yield of the anti-G17-Gly scFv, nevertheless, in terms of interaction, it showed statistically significant interactions with buffer’s pH (Fig. 4b, *p* < 0.029) and much stronger with Tris–HCl concentration (Fig. 4c*, p* < 0.009). Actually, 50 mM Tris–HCl improved the extraction performance more efficiently when the concentration of EDTA lowered to 0.1 mM. In agreement with our results, Nossal *et.al*. found that the selective release of enzymes from the periplasm of exponentially cultured *E. coli* strains was great at 0.1 mM EDTA in comparing with higher concentrations when used at the first stage of the osmotic shock method (Nossal and Heppel 1966). It was proposed that host cells in the exponential growth phase were more susceptible and so the integrity of the inner membrane was compromised by higher concentrations of EDTA. Conversely, in a study exploring the performance of different chemicals in the selective release of different recombinant proteins (i.e., Fab D1.3, alpha-amylase, and beta-lactamase) from *E. coli* periplasm was reported that increasing the EDTA concentration from 1 to 10 mM resulted in increasing the specific recovery yield (Jalalirad 2013). This controversial result, probably, is attributable to the different bacterial growth phases subjected to periplasmic extraction. Induction of the protein expression at higher cell density (OD600 of 10), in contrast to exponential-phase induction in the current study (OD600 of 0.9), leads to the extraction of periplasmic proteins at the early stationary phase where the cells are found to be more resistant to higher concentrations of EDTA which, in turn, make possible achieving an effective periplasmic extraction with fewer host proteins contamination.

The ANOVA results also showed that in all 30 experiments the Tris–HCl concentration and buffer's pH, as linear terms, had a negative effect on the functional recovery yield, indicating the lower values of Tris–HCl concentrations and buffer's pH improved the functional extraction yield of anti-G17-Gly scFv. As shown in Fig. 4d, the interaction model terms of Tris–HCl concentration and buffer’s pH were found to be highly influential on the extraction of anti-G17-Gly scFv. In other words, the reduction of the Tris–HCl concentration increased the functional recovery yield of scFv, particularly when accompanied by decreasing the buffer’s pH. The reduced binding activity of anti-G17-Gly, as a consequence of disturbing its structural integrity and/or dilution of the scFv-containing periplasmic fraction with host cell proteins due to the partial cell lysis, would be the potential explanations for the adverse effects of the higher values of Tris–HCl and buffer’s pH on the functional recover yield. These results are consistent with the study of French C. et al*.*, where the specific activity of alpha-amylase (termed here functional recovery yield) recovered from the periplasm of *E. coli* improved upon decreasing the concentration of Tris–HCl from 200 to 10 mM in a combined lysozyme/osmotic shock fractionation method (French et al. 1996). It was stated that the cell viability could be compromised by the high concentration of Tris–HCl (200 nM) and a high pH value of 8, and so the functional recovery yield of alpha-amylase decreased owing to the presence of contaminating cytoplasmic proteins. There are also controversial reports on the improvement of extraction efficiency of periplasmic proteins thorough exposing to higher values of Tris–HCl and buffer’s pH (Neu and Heppel 1964, 1965; Rathore et al. 2003), however, in these studies, the percentage of the total recovery yield was considered as a response rather than the specific recovery yield which is normalized by total protein, thereby specifying the extraction yield recovered only from periplasmic fraction.

In conclusion, the design of experiment based on RSM, which is a helpful technique for analyzing the effects of different variables in linear and interaction terms through avoiding a large number of laborious experiments, was applied to investigate a generic and efficient condition for the TES extraction of periplasmic scFvs. The results showed that Tris–HCl concentration and Buffer’s pH, and interaction between them were the main effective variables involved in the TES extraction method of scFv antibody fragments. In addition, the model developed on central composite design revealed that 50 mM Tris–HCl at pH 7.2, 0.1 mM EDTA, and induction time of 60 min, could be the optimal TES extraction condition in the functional recovery yield of scFv antibody fragments from periplasmic space of HB2151 at exponential growth phase after 5 h induction. This generic optimum condition is the true case when *E. coli* strain HB2151 as a recombinant host at the exponential growth phase is used for the production of periplasmic scFv antibody molecules and it can be altered depending on growth phase and type of strain. Hence, it will be worthy to study optimal conditions for TES extraction in different strains and growth phases.

## Data Availability

All data and materials are available from the corresponding authors on reasonable request.
